# Supplementary material for: Method Validation and Characterization of the Associated Uncertainty for Malondialdehyde Quantification in Exhaled Breath Condensate
Source: Antioxidants (Basel). 2021 Oct 22;10(11):1661. doi: 10.3390/antiox10111661 (PMC8615247; doi:10.3390/antiox10111661)
Supplement: Supplementary file 1 [file antioxidants-10-01661-s001.zip › antioxidants-1411048-supplementary.pdf]

antioxidants

Supplementary Information

Quantification of Malondialdehyde in Exhaled Breath Condensate: Method Validation and Characterization of the Associated Uncertainty

Maud Hemmendinger[1*], Jean-Jacques Sauvain[1], Nancy B. Hopf[1], Pascal Wild[2], Guillaume Suárez[1] and Irina Guseva Canu[1]

1 Department of Occupational and Environmental Health, Center for Primary Care and Public Health (Unisanté), Epalinges-Lausanne, Switzerland; jean-jacques.sauvain@unisante.ch (J.J.S.); nan-cy.hopf@unisante.ch (N.H.); guillaume.suarez@unisante.ch (G.S); irina.guseva-canu@unisante.ch (I.G.C.)
2 National Research and Safety Institute (INRS), Vandœuvre-lès-Nancy, France; pascal.wild@inrs.fr
* Correspondence: maud.hemmendinger@unisante.ch; Tel.: (+41 21 314 74 59)

Additional information on the calculation of the validation parameters

Limit of Detection (LOD) was calculated as 3 x intercept error on the slope of the calibration curve for samples. It was calculated over 12 days in accordance with [1].

Limit of quantitation (LOQ) is the smallest concentration at which the analyte can be quantified reliably, with an observed bias and imprecision smaller than an acceptable maximal deviation set at 20 %. The LOQ was calculated by multiplying the LOD with a factor of 3.

Precision

The method precision was determined by analyzing in quintuplicate, five levels of EBC QC, and five different EBC QC (corresponding to the intra-day precision). This measurement was repeated three non-consecutive days over a two-week period (corresponding to the inter-day precision), as described in the FDA/ICH guidelines [2]. The precision were examined as the intra-day precision (repeatability) and the inter-day precision (intermediate precision) following an ANOVA-based variance decomposition and expressed as relative standard deviation (% RSD) values (equation (1)).

$$\text{Method precision} = \sqrt{\text{Sr}^2 + \text{Sb}^2} \quad (1)$$

Repeatability (Sr) (% RSD) was calculated as square root of the repeatability variance corresponding to the sum of daily variances divided by the number of days (equation (2)).

$$Sr^2 = \frac{\sum_{i=1}^{n} Si^2}{n} \quad (2)$$

where Si^2 is the daily variance, n the number of days.

The intermediate precision (Sb) (% RSD) was calculated as the square root of the inter-daily variance corresponding as the mean of the daily variances minus the repeatability variance divided by the number of replicates (equation (3)).

$$\text{Sb}^2 = s(\overline{zi})^2 - \frac{Sr^2}{r} \quad (3)$$

where, $\text{s}(\overline{\text{zi}})^2$ is the variance of daily averages, r the number of replicates.

Matrix effects

The matrix effect was evaluated by comparing the slopes of the calibration curves obtained in water and in EBC, using QC samples across different analytical batches. An unpaired t-test was used for statistical comparison. A p value of 0.05 was used as the cut-off for significance.

Recovery rate

The recovery rate was calculated as the mean of quintuplicate QC solutions obtained on the validation process divided by the expected concentration values for each level (n=5). The recovery rate was expressed as percentage values (% RR) [2] (equation (4))

$$Recovery\ rate\ \% = \frac{\text{practical concentration QC at level i}}{\text{Theoretical concentration QC at level i}} * 100\% \quad (4)$$

Accuracy

Accuracy was calculated as the relative back calculated concentrations (evaluated from the calibration curve), with respect to their targeted concentrations (expressed in percentage of variation from the targeted concentration) with an acceptance limits of bias set at 20 % [3].

Storage

The stability of the MDA-DNPH derivative at room temperature (23 °C) was studied by reanalyzing samples kept in the auto-sampler after 24 or 48h. QC solutions and non-spiked EBC sample were also re-analyzed after 8 months storage at - 80 °C.

Uncertainty

Quantification of the uncertainty components at LOQ (209 pg/ml):

Based on validation data, three major contributions to uncertainty and their relative uncertainties are listed in the table below :

Description	Value x	Standard uncertainty u(x)	Relative standard uncertainty u(x)/x	Comments
Bias/recovery	0.924	0.037[a]	0.040	Based on validation data (n=15)
Precision	1.0	0.11[b]	0.110	Based on validation data (n=15)
Purity	1.0	0.023[c]	0.023	Indication on the bottle
	**u_{total} (x)**		0.11	
	**Coverage factor (95%)**		2	
	**Expanded uncertainty**		**0.23**	

[a] : In the validation procedure, a value of 92,4 % was found with a deviation standard (s) of 13 %. The standard uncertainty was calculated as : s $/\sqrt{n}$.

[b] : the standard uncertainty was calculated as the combination of repeatability and intermediate precision.

[c] : the standard uncertainty was calculated as 4% of impurity (given by the producer) divided by $\sqrt{3}$ (rectangular distribution).

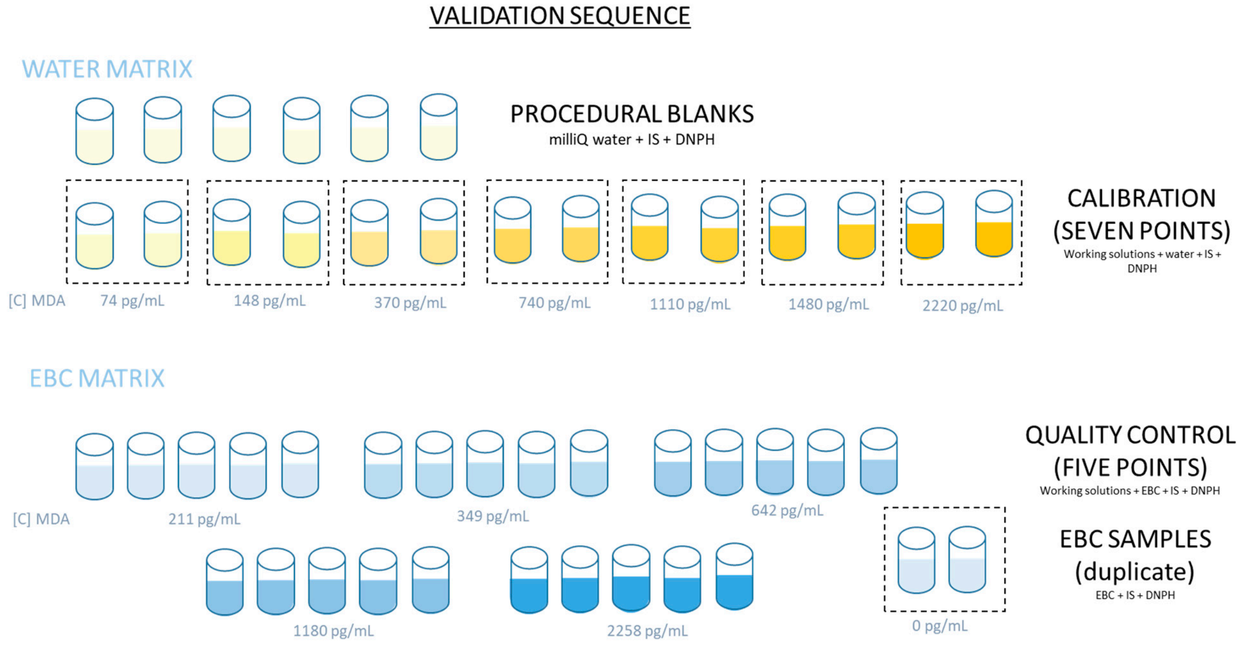

Figure S1. Validation sequence in three different days.

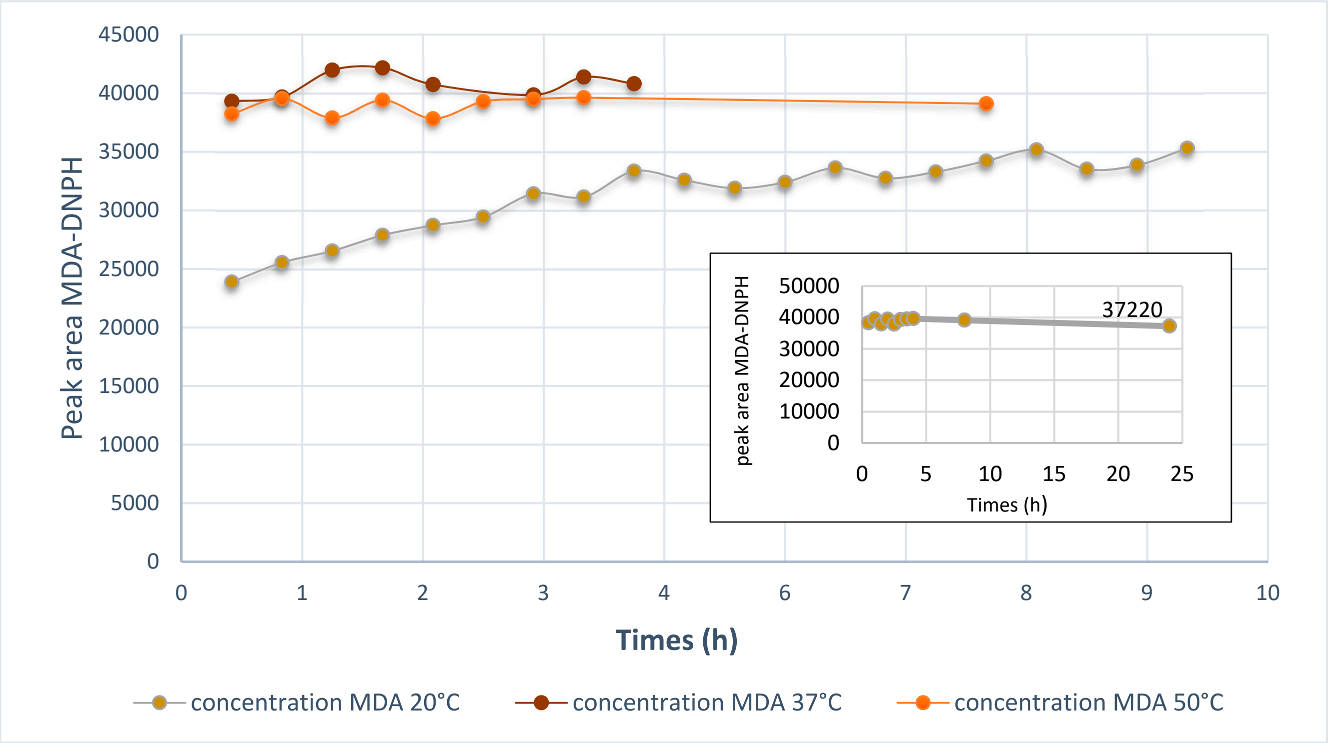

Figure S2. Effect of the reaction temperature on the MDA-DNPH formation as a function of time. The small box represents the variation of the MDA concentration at 20°C over a period of 24 hours

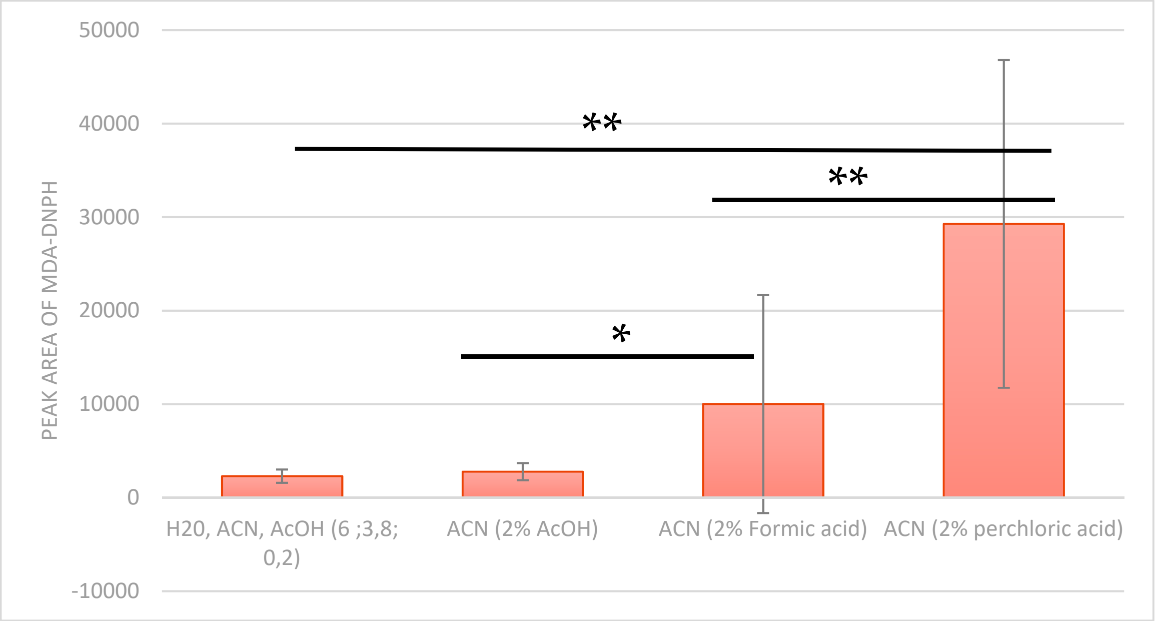

Figure S3. Contribution of the acid type on the signal of MDA-DNPH in procedural blanks. The error bars correspond to the standard deviation of five measurements. Comparisons were made by one-way ANOVA followed by Tukey's test (*$p<0.05$; **$p<0.005$). AcOH: acetic acid; ACN: acetonitrile.

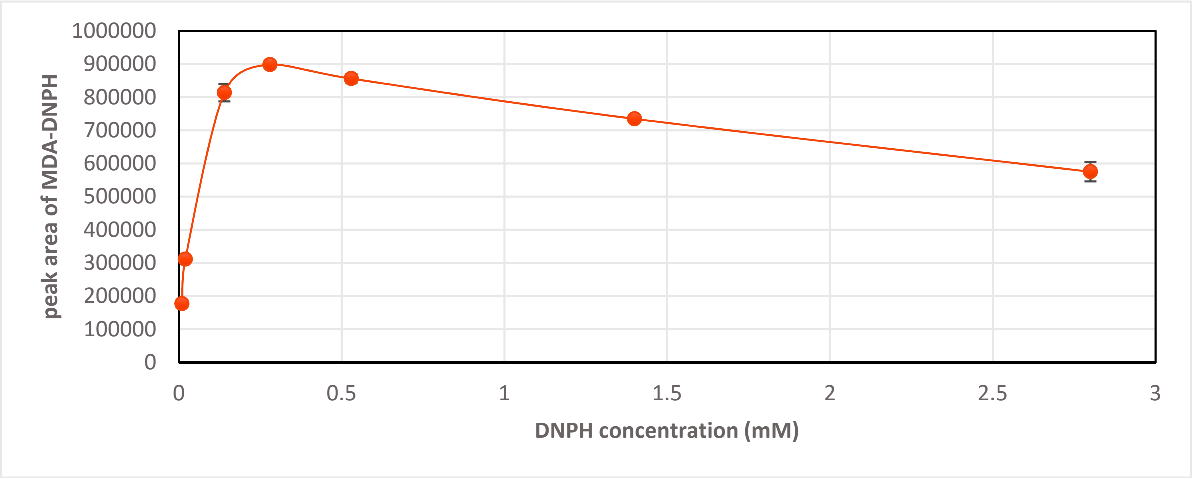

(a)

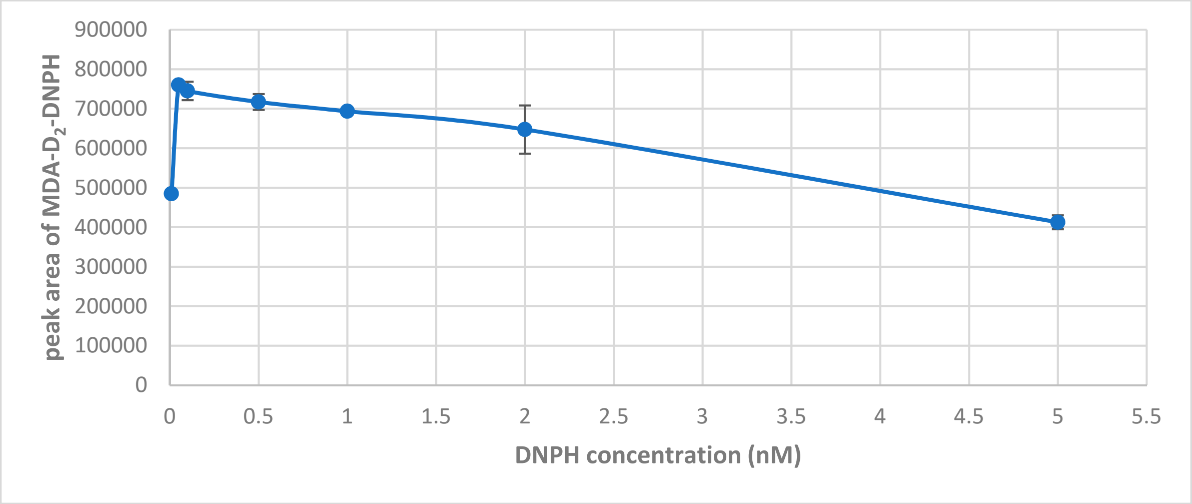

(b)

Figure S4. Effects of the added DNPH concentration expressed in mM on a) the peak area of MDA-DNPH b) the peak area of MDA-d2-DNPH for a standard solution at 35 ng/ml. The error bars correspond to the standard deviation of three measurements.

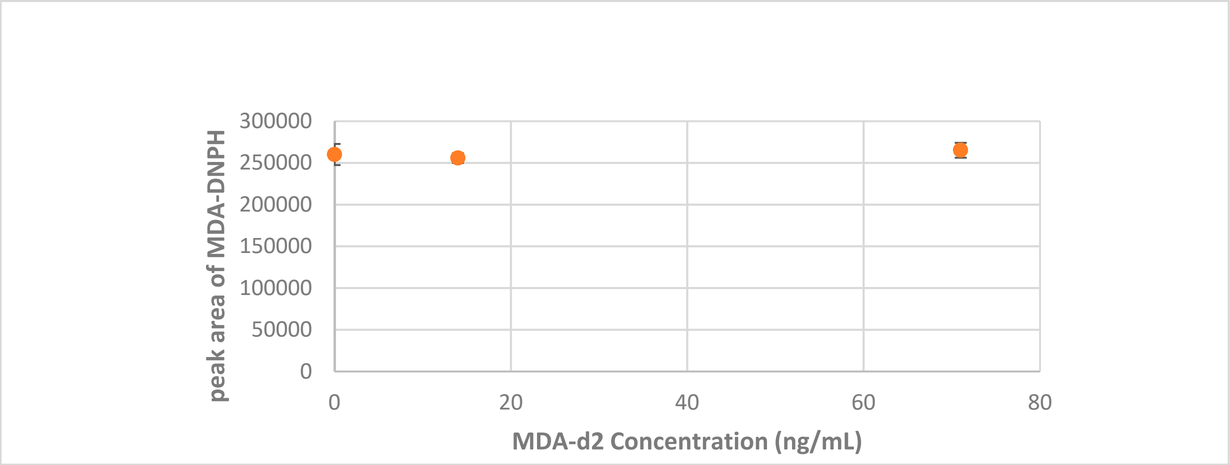

Figure S5. Effect of MDA-d2 concentration on the peak area of MDA-DNPH. For a standard solution at 0 ng/ml. The error bars correspond to the standard deviation of four measurements.

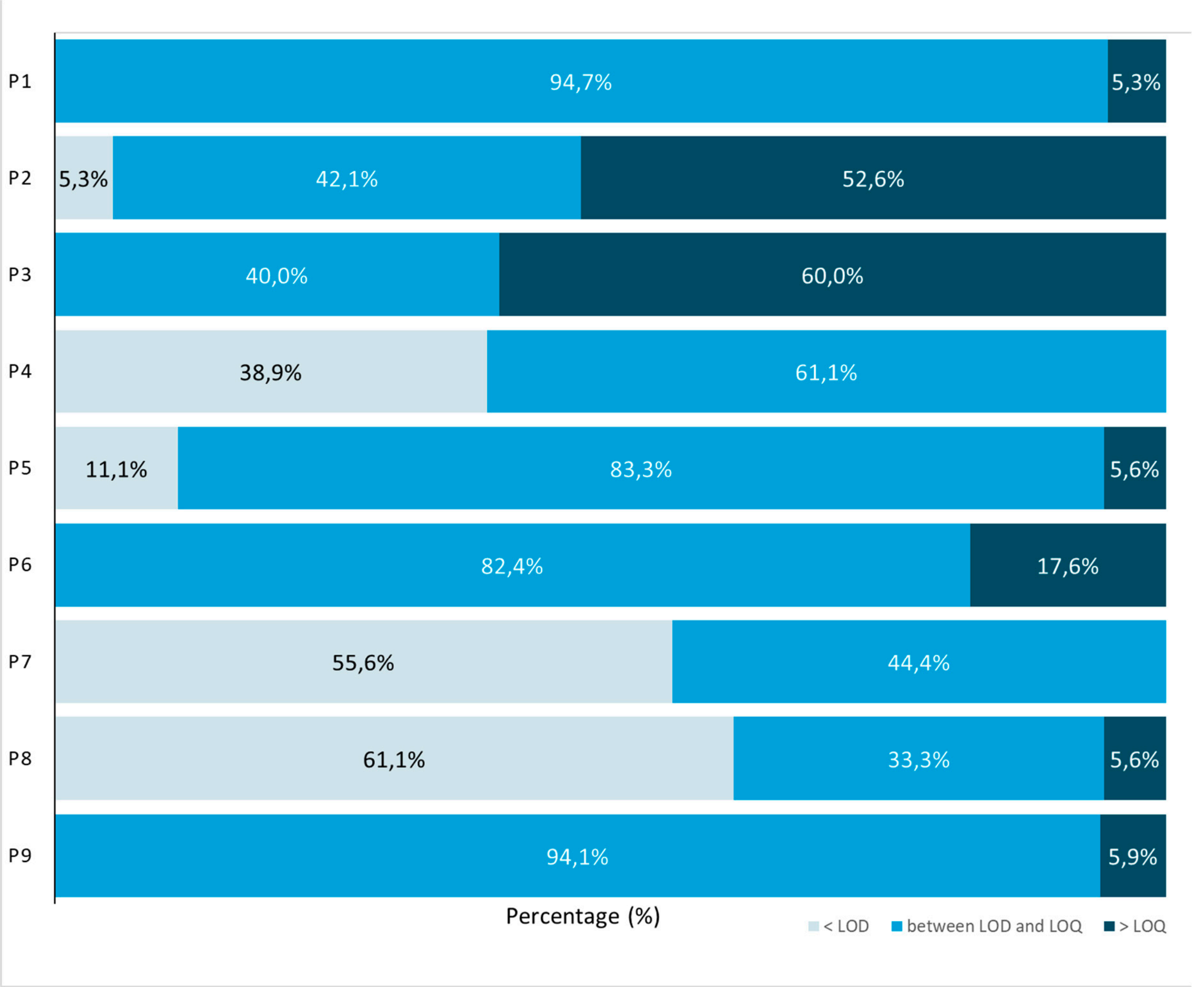

Figure S6. Stacked bar graph representing the percentage of values per participant below the LOD (light blue), between the LOD and LOQ (blue) and above the LOQ (dark blue).

References

1. Anderson, D.J. Determination of the lower limit of detection. *Clinical Chemistry* **1989**, *35*, 2152-2153, doi:10.1093/clinchem/35.10.2152.
2. FDA, U.S. Bioanalytical Method Validation Guidance for Industry. *United States Food & Drug Administration* **2018**, 44.
3. Boulanger, B.; Chiap, P.; Dewe, W.; Crommen, J.; Hubert, P. An analysis of the SFSTP guide on validation of chromatographic bioanalytical methods: progress and limitations. *Journal of pharmaceutical and biomedical analysis* **2003**, *32*, 753-765, doi:10.1016/s0731-7085(03)00182-1.
